# Refractory hypoglycemia induced by a duodenal wall gastrointestinal stromal tumor: A case report

**DOI:** 10.22088/cjim.12.0.447

**Published:** 2021

**Authors:** Foolad Eghbali, Reza Karami

**Affiliations:** 1 Minimally Invasive Surgery Research Center, Iran University of Medical Sciences, Tehran, Iran; 2 Center of Excellence of International Federation for Surgery of Obesity, Hazrat-e-Rasool Hospital, Tehran, Iran

**Keywords:** Hypoglycemia, GIST, IGF-II

## Abstract

**Background:**

Tumor-associated hypoglycemia can be caused by non-islet cell tumors including gastrointestinal stromal tumor (GIST) which is a rare paraneoplastic syndrome that leads to the release of insulin-like growth factor-2 (IGF-2).

**Case Presentation:**

We report the case of a 45-year old woman who was admitted to our hospital with refractory hypoglycemic episodes. We found normal serum insulin and c-peptide level and abdominal CT-scan showed a small duodenal wall lesion suggesting insulinoma. After tumor resection, hypoglycemia symptoms were recovered, but the pathological findings demonstrated the lesion was GIST.

**Conclusion:**

In a small gastrointestinal lesion with hypoglycemic symptoms we should consider IGF-II secreting GIST in addition to insulinoma.

Hypoglycemia is a common event confronted in clinical practice and is usually presented simultaneously with the treatment of diabetes mellitus. This condition can be induced by insulin or other antidiabetic agents (1), though a little number of hypoglycemic cases are spontaneous (2). Hypoglycemia can also be arising from adrenal or hepatic diseases, tumors, hypocortisolism, etc (1). Tumor-associated hypoglycemia can be caused by islet cell tumors or non-islet cell tumors. Insulinomas are tumors originating from pancreatic islet cells causing insulin hypersecretion, resulting in hypoglycemia. Non-islet cell tumor hypoglycemia (NICTH) is a rare paraneoplastic syndrome that causes the release of insulin-like growth factor-2 (IGF-2) and may be induced by different types of tumors including hepatocellular carcinoma, lymphoma, and mesenchymal tumors (3). The most prevalent mesenchymal tumor originating from gastrointestinal tract is gastrointestinal stromal tumor (GIST) which is positive for CD117 receptor in immunohistochemical evaluations (4). Its annual incidence is reported as 5-15 cases per one million and consists about 2% of all GI tract neoplasms. The most frequent GIST presenting location is the stomach, followed by the small intestines. The duodenum is an uncommon site for GIST and accounts for about 3-5 percent of all GIST cases. The most prevalent symptom of GIST is gastrointestinal bleeding (4). Hypoglycemia is a rare manifestation of GIST which only a few cases have been reported so far. NICTH incidence is estimated about one per million person and no exact data is indicating how many cases are reported so far (5). The treatment of choice for GIST is surgical excision. In large gastric tumors, total gastrectomy may be needed (6). Almost all the patients will be completely cured after lesion removal (3). This case report describes a patient with a duodenal wall GIST who underwent the lesion surgical removal and got free from hypoglycemic episodes.

## Case presentation

We report the case of a 45-year-old woman who was referred to our hospital for evaluation after some episodes of hypoglycemia neurological symptoms including weakness, fatigue, dizziness, blurred vision, headache, improving after ingestion of sweetmeat and simple carbohydrates but not including loss of consciousness, confusion, bowel habit change, and seizure. In addition, a low serum glucose level was documented in the last episode (47 mg/dL; reference range: 70-95 mg/dL).

 Her symptoms began 2 months before admission and almost occurred twice a month. The episodes often occurred in fasting time and occasionally after food consumption. Her blood glucose was in the range of 40 to 70 mg/dl during hypoglycemic episodes. 

She had about 20kg weight loss during the last 3 months despite increased food intake and also suffered from abdominal epigastric pain. She had no significant past medical history and any medications without any point in family history. The physical examinations and vital signs had no abnormal finding. Due to persistent neuroglycopenia symptoms despite oral sweat foodstuff consumption, she was prescribed D/W 10% (Dextrose Water) serum to relief the symptoms. Her complete blood cell count, electrolytes, BUN, Cr and liver function tests were normal. Other laboratory tests are placed in table 1.

**Table1:** The laboratory findings of the patient on hospital admission

**Test**	**Result**	**Normal Range**
BS	48	70-110mg/dl
C-Peptide	1.1	1-3.5 ng/ml
Insulin	10.3	2.5-25 mIU/ml
8 Am Cortisol	10.8	9-25 mcg/dl
Hb A1C	4.5	<5.7%
TSH	4.42	0.28 – 4.3 mIU/ml
Total T4	5.58	4.9 – 13.9 mcg/dl

**Fig. 1:**
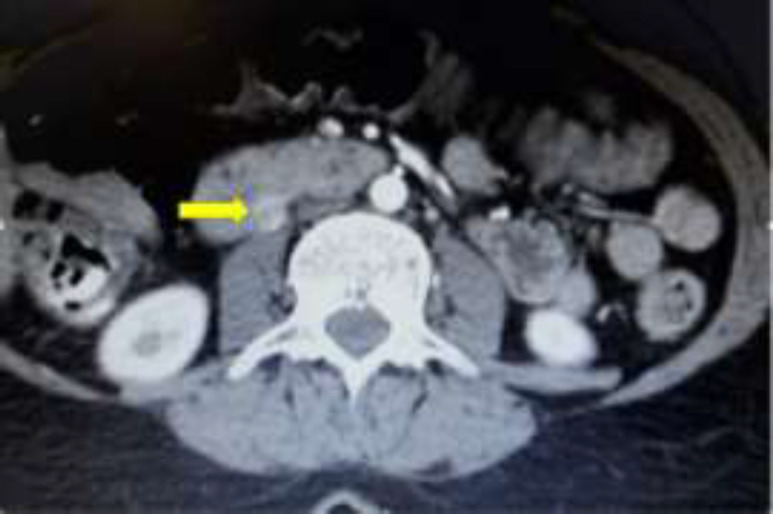
Nodular lesion in the posterior wall of third duodenal in abdominopelvic CT scan

According to fasting hypoglycemia and Whipple tirade (fasting hypoglycemia<50, hypoglycemia symptoms and immediate symptom relief with glucose consumption), insulinoma was strongly considered. For lesion localizing, a triphasic abdominopelvic CT scan was accomplished. The finding was a 10*12mm nodular lesion in the posterior wall of the third duodenal portion anterior to psoas muscle in the level of third lumbar vertebrae which had strong enhancement in arterioportal phase (fig. 1). There was no other significant finding. In the endoscopic ultrasonography, there was no pathology seen in the pancreas and biliary tract. 

**Fig. 2:**
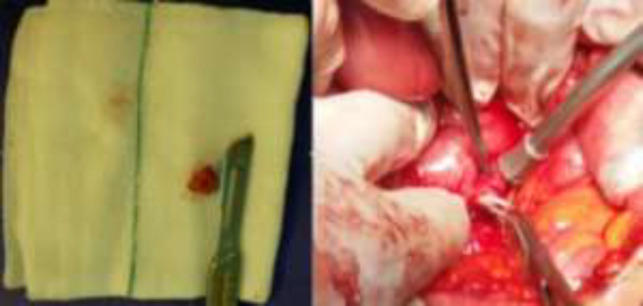
Third portion duodenal wall lesion estimated 12mm

All of the evaluations indicated that a duodenal wall lesion is the reason for hypoglycemia presence. Surgical treatment was scheduled. During exploratory laparotomy after Kocher maneuver, a round shape, firm consistency lesion 12mm diameter in the third portion of duodenum originating from the seromuscular layer (fig. 2) was found which excised by enucleation without mucosal injury. Immediately after resecting the lesion, the blood sugar level was placed in the normal range. The patient spent her postoperative time without any complications and was discharged on the third day after surgery. In a 6-month follow-up, she did not have any hypoglycemic episodes.

**Fig 3:**
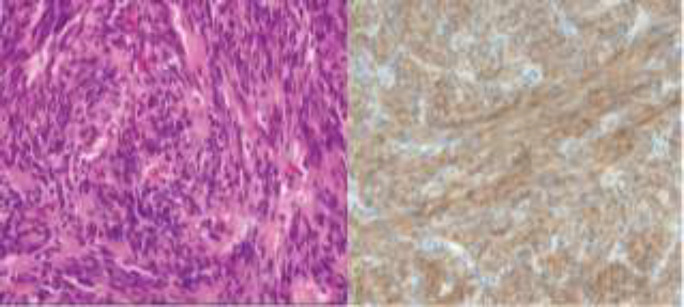
hematoxylin and eosin staining (left) 40X magnification image of a GIST; (right) immunohistochemistry view indicating spindle cells positive for CD117.

In pathologic study, low grade gastrointestinal stromal tumor with spindle cell type and 1/50 HPF mitotic rate without necrosis was reported (fig. 3). In immunohistochemistry assessment, CD117 was positive in tumoral cells (fig. 3), SMA and S100 were negative while CD34 was patchy positive and 2% of the tumor cells being positive for the proliferation marker Ki67. 

## Discussion

Symptomatic hypoglycemia which causes neurologic signs and symptoms, is called neuroglycopenic signs. These signs and symptoms are generally nonspecific, on the other hand, it is impossible to define an accurate plasma glucose level that leads to neuroglycopenia. Therefore, hypoglycemia is confirmed by documentation of the Whipple’s triad which defines symptomatic hypoglycemia, consisting of signs and symptoms of hypoglycemia, a document of low plasma glucose level and terminated signs and symptoms after increase in blood sugar level (7). 

The most prevalent etiology of hypoglycemia is alcohol abuse, using drugs affect plasma glucose level sepsis or liver dysfunction. Hypoglycemia can be also induced by different metabolic disorders (8). As Murad MH et al. recommended in a systematic review, in the same direction of this study, we measured serum cortisol level at 8 A.M which had no change in value (8).

Another uncommon etiology of hypoglycemia is tumoral lesions. The most widespread etiology is insulinoma. Other pathologies, can either induce hypoglycemia. The category of extra pancreatic tumors inducing hypoglycemia is termed as non-islet cell tumor with hypoglycemia (NICTH). NICTH is a rare paraneoplastic syndrome with an estimated incidence of nearly 1/1,000,000 person-years (5). NICTH is usually originated from gastrointestinal system, but can be discovered in thoracic cavity, kidney and adrenal gland (9). 

It has been reported that GIST can rarely represent hypoglycemic episodes which this feature is a rare manifestation (3). These cases of GIST induced hypoglycemia (GISTH) may be considered as subgroup of NICTH. The pathogenesis of GISTH has not been understood completely yet. Insulin-like growth factor II (IGF II), multiple liver metastases, bulking tumors and autoimmunity are the probable mechanisms leading to GISTH (10). It has been demonstrated that IGF II gene over-expression in neoplastic tissues results in development and longer life of neoplastic cells. In these tissues IGF II over expression leads to production of “big” IGF II which is IGF II precursor. Finally, big IGF II results in hypoglycemia through decreased glucose production and increased peripheral glucose uptake (10).

As this case presented some rare clinical findings, it makes it unique. These findings are extra ordinal when we compare our case with the patients suffering from hypoglycemia inducing GIST that were previously reported (3, 4). This patient had normal serum levels of insulin and c-peptide which was in consistent with Dean K. et al.’s study (11). 

In our case, fasting hypoglycemic episodes terminated immediately after surgery and no longer hypoglycemic episodes occurred, on the other hand, our investigations showed a small single lesion leading to hypoglycemia, but in literature, this was in contrast with other reports as most of the studies reported a metastatic or a large tumor causing hypoglycemia. (10, 11). As the preoperative investigations, especially abdominal CT-scan findings, suggested that our patient is suffering from insulinoma, we had not checked serum IGF-II level. Thus in such a patient especially with normal serum insulin and c-peptide level, the IGF-II level should be checked, because IGF-II secretory GIST should be considered as a differential diagnosis of extrapancreatic insulinoma.

It is very difficult to explain the normal serum insulin level and hypoglycemia in this case based on mechanism of IGF, c-peptide, and insulin as unfortunately we did not check the IGF-II level. We think that hypoglycemia along with normal insulin levels might be due to the clinical stage, since our patient stood in the initial stages of GIST and almost all previously reported patients were in metastatic stages (3, 4). However, more patients should be evaluated to conclude that normal, high or low insulin levels are stage-dependent.

In conclusion Hypoglycemia inducing GIST is a type of NICTH which is reported rarely. The leading mechanisms include insulin-like growth factor II hypersecretion. The hypoglycemic symptoms often are presented in huge or metastatic GIST, but in our case, a single small GIST causes the symptoms. It was cured by resection. Thus in a small GI lesion with hypoglycemic symptoms, we should consider IGF-II secreting GIST in addition to insulinoma.
